# *Saccharomyces boulardii* promoters for control of gene expression in vivo

**DOI:** 10.1186/s12934-023-02288-8

**Published:** 2024-01-07

**Authors:** Carmen Sands, Karl Alex Hedin, Ruben Vazquez-Uribe, Morten Otto Alexander Sommer

**Affiliations:** Novo Nordisk Foundation Center for Biosustainability, Technical University of Denmark, 2800 Kgs. Lyngby, Denmark

## Abstract

**Background:**

Interest in the use of engineered microbes to deliver therapeutic activities has increased in recent years. The probiotic yeast *Saccharomyces boulardii* has been investigated for production of therapeutics in the gastrointestinal tract. Well-characterised promoters are a prerequisite for robust therapeutic expression in the gut; however, *S. boulardii* promoters have not yet been thoroughly characterised in vitro and in vivo.

**Results:**

We present a thorough characterisation of the expression activities of 12 *S. boulardii* promoters in vitro in glucose, fructose, sucrose, inulin and acetate, under both aerobic and anaerobic conditions, as well as in the murine gastrointestinal tract. Green fluorescent protein was used to report on promoter activity. Promoter expression was found to be carbon-source dependent, with inulin emerging as a favourable carbon source. Furthermore, relative promoter expression in vivo was highly correlated with expression in sucrose (*R* = 0.99).

**Conclusions:**

These findings provide insights into *S. boulardii* promoter activity and aid in promoter selection in future studies utilising *S. boulardii* to produce therapeutics in the gut.

**Supplementary Information:**

The online version contains supplementary material available at 10.1186/s12934-023-02288-8.

## Background

The microbiome has increasingly been identified as a potential target for therapeutic interventions, due to its role in the development of a range of diseases [1–3]. One approach to modify the microbiome involves the application of synthetic biology tools to engineer microbes for therapeutic applications. These living, engineered microbes, termed advanced microbiome therapeutics (AMTs), can produce peptides or small molecules with therapeutic activity directly in the gastrointestinal tract. The yeast *Saccharomyces boulardii* has had a long history of safe use as a probiotic [4] and many of genetic tools originally developed for *S. cerevisiae* have been successfully adapted for use in *S. boulardii* [5–9]. Furthermore, as a eukaryotic organism, *S. boulardii* has the ability to perform more complex post-translational modifications on peptides and proteins [10], making it an attractive AMT chassis.

In the context of developing novel AMTs, the presence of a consistent and reliable expression system becomes crucial [11]. In particular, the expression of biosynthetic pathways and sensing circuits rely on the ability to balance pathway components using promoters of different strengths [12]. Therefore, a library of well-characterised promoters is needed for the establishment of complex therapeutic production in *S. boulardii*. The activities of select *S. cerevisiae* promoters have become well-characterised under laboratory conditions [13–16] as well as under industrially relevant conditions, such as oxygen limitation [17] and heat stress [18, 19]. While there are high levels of correlation between promoter activities in *S. cerevisiae* and *S. boulardii*; there is some divergence in certain cases. For instance, P_*ALD6*_ is significantly stronger in *S. boulardii* than in *S. cerevisiae* [6]. The markedly different expression under laboratory conditions and industrially relevant ones demonstrate the importance of environmental context in performing promoter characterisations. Studies with *E. coli* have identified promoters behaving similar in vitro and in vivo [20], yet expression of some promoters can vary greatly between in vitro and in vivo conditions [20] as well as along the gastrointestinal tract [21] as a result of the changing conditions across the GI tract [22–24]. Despite this, the use of synthetic biology tools often relies heavily on genetic parts that have only been characterised under optimal laboratory conditions, not those found in the gastrointestinal tract. Currently, only one study has characterised promoter expression in *S. boulardii* in vitro and used these promoters to successfully express complex biosynthetic pathways in vivo [6]. Yet, detailed characterisation of *S. boulardii* promoters in the gastrointestinal tract is needed to establish promoters that can be reliably used for in situ expression of complex biosynthetic pathways.

Direct characterisation of promoters in the rodent gastrointestinal tract is the gold standard when it comes to understanding how promoters behave in vivo; however, animal work comes with economic, technical and ethical barriers [25]. In vitro approaches, where gastrointestinal conditions are replicated in the lab, present a more accessible but potentially less accurate alternative [25]. That said, the translatability of in vitro approaches for promoter characterisation has not yet been investigated for *S. boulardii*. In this study we develop in vivo and in vitro promoter characterisation protocols, using GFP as a tool to evaluate the performance of 12 *S. boulardii* promoters, to compare the translatability of various in vitro conditions. Our in vitro characterisation compares promoter expression under conventional conditions (aerobically, with glucose as a carbon source) and with conditions more relevant to the gastrointestinal tract (namely, carbon sources found in gut or diet, and anaerobic conditions). Finally, this work aims to provide researchers working with *S. boulardii* a library of promoters that have been thoroughly characterised in various carbon sources, and in the murine gastrointestinal tract.

## Results

### Promoter selection

**Fig. 1:**
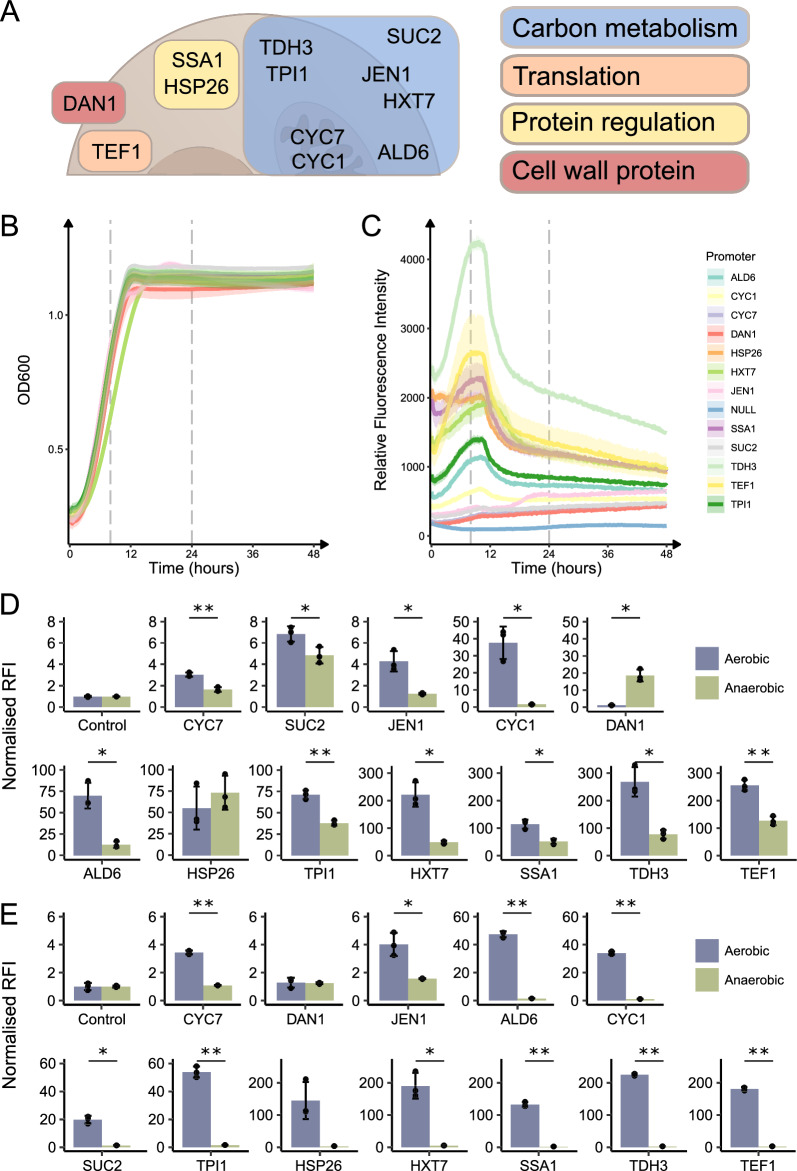
Promoter function and characterisation in glucose. **A** Schematic overview of native roles of promoter genes. **B** Aerobic growth of the promoter strains over time. **C** Aerobic relative fluorescence intensity (RFI) of yEGFP produced by the promoter strains over time. Dashed lines in (**B**) and (**C**) indicate 8 and 24 h, the time points that were selected for further investigation by flow cytometry. **D** Mean of the normalised median relative fluorescence intensity of yEGFP from the 8 h time point and (**E**) from the 24 h time point of the aerobic (blue) and anaerobic (green) flow cytometry experiments. All plots display the mean of three biological replicates, from independent pre-cultures. Bar plots are displayed as the mean ± SD. Points represent individual replicates. * p < 0.05 and ** p < 0.005 (**D**) and (**E**) were analysed with t-tests and p-values were adjusted for false discovery rate

Suitable candidate promoters were identified via a literature search. We focused on literature of *S. cerevisiae* promoters, due to the lack of literature on *S. boulardii*, knowing that there should be a high level of transferability between the two species [6]. Promoters were selected that met at least one of the following two criteria: it is in widespread use in synthetic biology applications, or its expression is dependent on conditions present in the gastrointestinal tract (such as low glucose or oxygen levels). On this basis, we selected twelve *S. boulardii* promoters for this study: P_*ALD6*_, P_*CYC1*_, P_*CYC7*_, P_*DAN1*_, P_*HSP26*_, P_*HXT7*_, P_*JEN1*_, P_*SSA1*_, P_*SUC2*_, P_*TDH3*_, P_*TEF1*_ and P_*TPI1*_. All the sequences used had > 97% identity to those of *S. cerevisiae* S288C as determined by BLAST search (Table 1). The rationale for selecting each promoter is summarised in Table 1. The selected promoters are involved in diverse areas of cellular metabolism; however, those involved in carbon metabolism are overrepresented (Fig. 1A; Table 1). Due to the lack of available glucose in the lower sections of the gastrointestinal tract [26] we aimed to select promoters that do not require high levels of glucose for activity, hence the over representation of promoters involved in carbon metabolism.

**Table 1:** Summary of native promoter functions and rationale for inclusion in the study

Promoter	Native gene function	Reasoning for selection	% identity to S288C
P_*ALD6*_	Cytosolic aldehyde dehydrogenase; conversion of acetaldehyde to acetate	Involved in growth on non-fermentable carbon sources. Evidence for stronger expression in S. boulardii than S. cerevisiae	99.68
P_*CYC1*_	Cytochrome c isoform 1. Electron carrier during cellular respiration	Common medium expression promoter	98.75
P_*CYC7*_	Cytochrome c isoform 2. Electron carrier during cellular respiration	Similar function to CYC1, but induced in hypoxic conditions	97.86
P_*DAN1*_	Cell wall mannoprotein	No expression in presence of oxygen	99.36
P_*HSP26*_	Heat shock protein. Chaperone that prevents aggregation of unfolded proteins	High expression in response to heat shock/carbon starvation/stress	97.50
P_*HXT7*_	High-affinity glucose transporter	Low/no expression in presence of glucose. High expression in absence of glucose. Note: low oxygen can lead to reduced expression	99.90
P_*JEN1*_	High-affinity uptake of monocarboxylate carbon sources (e.g. lactate, acetate)	Repressed at high glucose. Expressed in presence of alternative carbon sources e.g. lactate, glycerol and acetate	97.43
P_*SSA1*_	Multifunctional ATPase involved in many aspects of protein regulation	Constitutive promoter. Low expression during exponential growth phase; high expression in post-diauxic phase	99.33
P_*SUC2*_	Invertase, has secreted and intracellular forms. Hydrolyses sucrose to glucose and fructose	High expression in presence of sucrose	98.90
P_*TDH3*_	Glyceraldehyde-3-phosphate dehydrogenase, involved in glycolysis and gluconeogenesis	Strong ‘constitutive’ promoter	98.97
P_*TEF1*_	Translational elongation factor EF-1 alpha; delivers charged tRNAs to the ribosome	Strong ‘constitutive’ promoter	99.52
P_*TPI1*_	Triose phosphate isomerase, involved in glycolysis	Common promoter for protein production. Medium strength	99.86

### Characterisation of promoters under aerobic and anaerobic conditions using yEGFP as reporter

We began by characterising the promoters in glucose under aerobic and anaerobic conditions. This allowed us to test if the *S. boulardii* promoters behave similarly to their homologues in *S. cerevisiae* and to test if our protocol could be used to characterise expression under anaerobic conditions. We chose yeast enhanced green fluorescence protein (yEGFP) [27] as our measure of expression as it has previously been shown to be a good reporter for gene expression [13]. Although yEGFP requires molecular oxygen to fluoresce [28], it has a short maturation period once exposed to oxygen [29]. While this precludes continuous fluorescence measurements, defined time points can be analysed by including an aerobic incubation step [20]. This is desirable as yEGFP is brighter than anaerobic variants, which have primarily been designed for fluorescence imaging [30]. It is therefore better at resolving small differences in expression, particularly for low expression promoters. We integrated the promoter-yEGFP expression cassettes at the XII-5 locus [31], which has previously been used for the expression of therapeutic peptides [8]. Additionally, we generated a control strain with a negative integration (P_*null*_) at the same site. In total, we generated 13 strains for characterisation.

To determine which time points to analyse in depth, we first measured growth and fluorescence of biological triplicates continuously for 48 h aerobically with a microplate reader. The P_*HXT7*_ strain was the only strain to have a statistically significant difference in growth rate relative to all other strains, with a reduced maximum growth rate (Fig. 1B and Additional file 2: Table S1). Promoters P_*ALD6*_, P_*CYC1*_, P_*HXT7*_, P_*SSA1*_, P_*TDH3*_, P_*TEF1*_ and P_*TPI1*_, accumulated fluorescence over the course of the exponential phase. P_*HSP26*_, had a steady level of fluorescence over the exponential phase (Fig. 1C). From here, we decided to focus on 8 h and 24 h. These time points cover exponential and stationary phase respectively, as well as significantly different fluorescence levels.

For analysis of the 8- and 24 h time points, we used flow cytometry to measure the relative fluorescence intensity. Cycloheximide was added to samples to prevent translation of yEGFP [32] once the sample has been taken, and a 20 min aerobic incubation step was included for all samples to allow yEGFP to mature before measurement. Aerobically at 8 h promoter expression can be ranked from highest to lowest as follows: P_*TDH3*_ > P_*TEF1*_ > P_*HXT7*_ > P_*SSA1*_ > P_*TPI1*_ > P_*ALD6*_ > P_*HSP26*_ > P_*CYC1*_ > P_*SUC2*_ > P_*JEN1*_ > P_*CYC7*_ > P_*DAN1*_ > P_*null*_. Promoter expression was overall lower in anaerobic conditions than in aerobic conditions. Anaerobically at 8 h promoter expression can be ranked from highest to lowest as follows: P_*TEF1*_ > P_*TDH3*_ > P_*HSP26*_ > P_*SSA1*_ > P_*HXT7*_ > P_*TPI1*_ > P_*DAN1*_ > P_*ADL6*_ > P_*SUC2*_ > P_*CYC7*_ > P_*CYC1*_ > P_*JEN1*_ > P_*null*_. P_*DAN1*_ was the only promoter with statistically significant higher expression (15-fold) in anaerobic conditions; P_*HSP26*_ was the only promoter with no statistical difference in expression between oxygen conditions (Fig. 1D). Expression was overall lower at 24 h than it was at 8 h; P_*HSP26*_ was the only promoter with higher expression at 24 h than 8 h (Fig. 1E). At 24 h, anaerobic expression was further reduced for most promoters; P_*DAN1*_ was no longer expressed beyond the level of the P_*null*_ control strain (Fig. 1E).

Overall, the relative expression levels correlate with previously published findings [13, 14, 16]. Additionally, the induction of P_*DAN1*_ under anaerobic conditions serves to confirm that our method for anaerobic characterisation is functional. With this confirmation, we decided to move forward to characterising our selected promoters under conditions more relevant to the gastrointestinal tract.

### Carbon source-dependent regulation of *S. boulardii* promoters

To represent the conditions of the gastrointestinal tract we prepared defined media with acetate, fructose, sucrose and inulin as carbon source. Acetate was chosen as the most common short chain fatty acid present in the colon [33], fructose and sucrose were chosen as common dietary carbon sources [34] and inulin was chosen as it is a common prebiotic that supports *S. boulardii* growth [35].

**Fig. 2:**
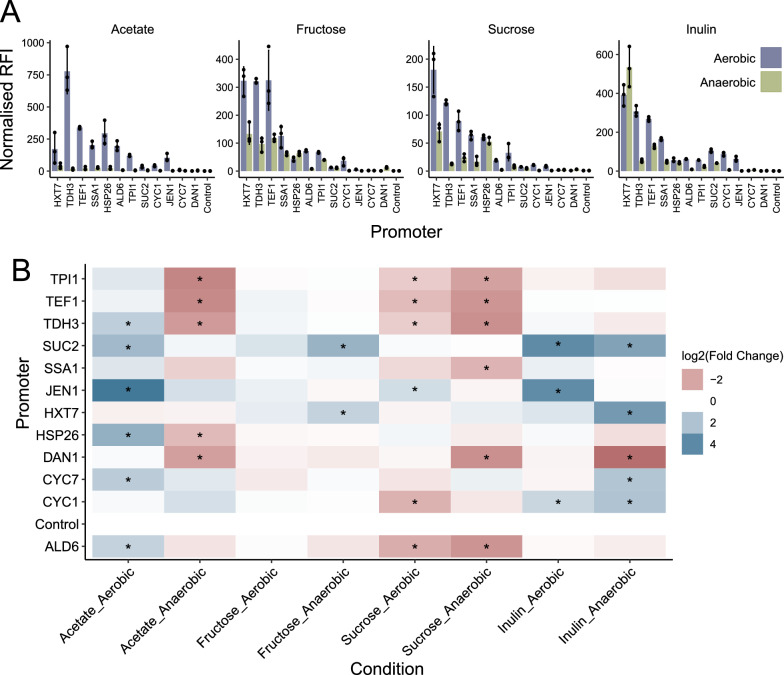
Promoter expression in simulated gut conditions. **A** Normalised median relative fluorescence intensity of yEGFP from the 8 h time point. Points represent the individual replicates and error bars represent the standard deviation. **B** Log2 of the fold change in normalised RFI relative to the level in glucose. All plots show the mean of three biological replicates from independent pre-cultures. Data was analysed with a one-way ANOVA and Dunnett’s post hoc test, with Control from the same Carbon_Oxygen condition as a reference. P-values were adjusted for all comparisons. * p < 0.05

All the characterised promoters experienced carbon-source dependant changes in expression (Fig. 2A). At 8 h, under aerobic conditions, P_*JEN1*_ was induced 24-fold in acetate and 15-fold in inulin (relative to its expression in glucose), making it the promoter with the highest induction by a non-glucose carbon source. P_*SUC2*_ was induced 15-fold in inulin but not induced in sucrose; this is possibly due to the 8 h time point falling relatively late in the exponential phase, and most of the sucrose having been consumed by this point (Fig. 2B).

Under anaerobic conditions, P_*TDH3*_, P_*TEF1*_ and P_*TPI1*_ were all significantly repressed in acetate and sucrose, relative to their expression in glucose (Fig. 2B). Additionally, P_*DAN1*_ had significantly lower expression in acetate, inulin and sucrose compared to glucose (Fig. 2B). Conversely, P_*CYC7*_, P_*HXT7*_ and P_*SUC2*_ showed significant induction in inulin relative to glucose. P_*HXT7*_ was induced 11-fold in inulin relative to glucose, producing a normalised RFI of 534 and thus the highest anaerobic expression measured in this study (Fig. 2B).

At 24 h, expression was generally similar or slightly lower than expression at 8 h, except for P_*HSP26*_, which has higher expression (Additional file 1: Fig S1). P_*JEN1*_ was the only promoter to experience significant carbon-source related induction under aerobic conditions, with 41 and 38-fold increases in expression in acetate and inulin respectively (Additional file 1: Fig S1). Under anaerobic conditions, P_*CYC1*_, P_*CYC7*_, P_*HSP26*_, P_*HXT7*_, P_*SSA1*_, P_*SUC2*_, P_*TDH3*_, P_*TEF1*_ and P_*TPI1*_ were all significantly induced in inulin. P_*HSP26*_, P_*HXT7*_, and P_*TEF1*_ had the greatest change with 53, 65 and 79-fold induction compared to glucose (Additional file 1: Fig S1).

Overall, promoter expression is similar in glucose and fructose, while sucrose appears to have a predominately negative effect on expression. Inulin has a predominately positive effect on expression levels, while acetate’s effect on expression is closely oxygen-dependent. Thus, carbon source has a significant effect on promoter expression, with even promoters considered constitutive being induced and/or repressed in some of the carbon sources considered here. To ensure that all the promoters we characterise in vivo have sufficient signal to be detected over the background fluorescence of the mouse gut content, we selected the strongest seven promoters, as well as P_*JEN1*_, the most carbon-inducible promoter.

### Characterisation of promoters in the murine gastrointestinal tract

**Fig. 3:**
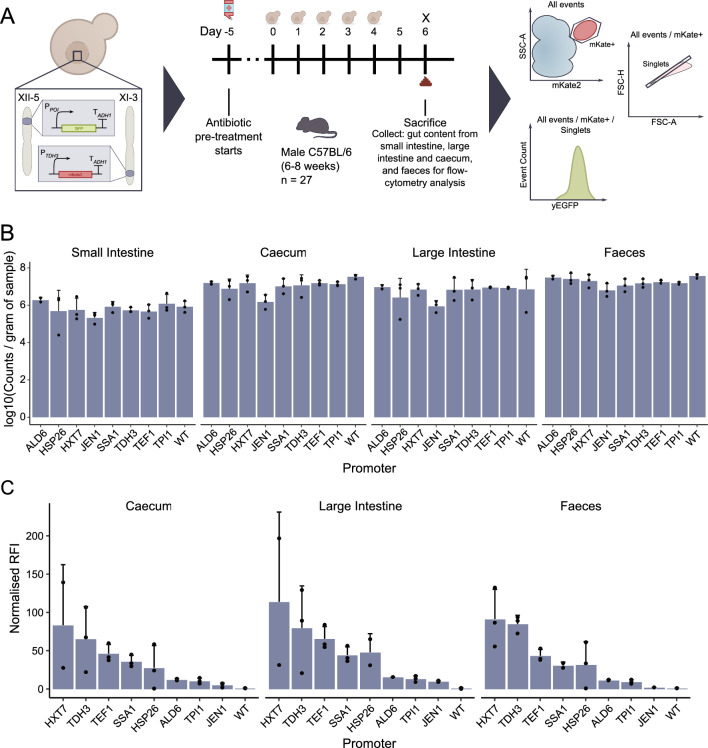
Characterisation of promoters in the murine gastrointestinal tract. **A** Schematic overview of the in vivo characterisation, representing the strain design, the animal study and the flow cytometry analysis. 27 mice were divided into 9 groups, with three mice per strain of *S. boulardii*. **B** Colonisation across the gastrointestinal tract, as determined from flow cytometry. **C** Normalised median relative fluorescence intensity of yEGFP across the gastrointestinal tract, from mKate2 + cells. Bar plots are presented as the mean + SD. Points represent the mean of two technical replicates. Faeces data shown here come from samples collected on day 6 of the study

To assess promoter expression levels in the murine gastrointestinal tract eight promoters (P_*ALD6*_, P_*HSP26*_, P_*HXT7*_, P_*JEN1*_, P_*SSA1*_, P_*TDH3*_, P_*TEF1*_ and P_*TPI1*_) and the control strain were selected. Previous studies have highlighted the difficulty in isolating target microbes from mouse gut content using flow cytometry, due to high levels of green background fluorescence [20]. Hence, we integrated an mKate2 [36] expression cassette at the XI-3 locus to allow target cells to be identified via the red channel, before green fluorescence was measured (Fig. 3A).

For the study, 27 mice were started on an antibiotic cocktail 5 days prior to the study starting [37]. This reduces their microbiome and improves *S. boulardii* colonisation in the gastrointestinal tract [37]. The dual red/green promoter strains were administered daily via oral gavage for a period of 5 days. A 48 h washout period was included prior to sacrificing the mice, during which *S. boulardii* was not administered. The washout period reduced the likelihood of measuring yEGFP from the gavage material and ensures the cells have sufficient time in the gastrointestinal tract to begin to adapt to their surroundings. Faeces were collected on days 1, 4 and 6 of the study; small intestine, caecum and large intestine samples were collected during dissection on day 6 (Fig. 3A). Dissection samples were homogenised in a solution containing 10 mg/mL cycloheximide, immediately after collection to ensure no new yEGFP was translated following sample collection.

The addition of cycloheximide (a fungicide) to the sample solution meant colonisation could not be estimated via colony forming units (CFUs). As an alternative to CFU, we decided to use the event count from the flow cytometer as a proxy for CFU. We considered every mKate2-positive event captured in the singlets gate (Fig. 3A) as equivalent to a single CFU, to reduce the likelihood of counting debris or double cells collected in the mKate2 + as a single CFU. Using this method, we found colonisation was highest in the faeces and lowest in the small intestine with 23.7 × 10^6^ and 1.1 × 10^6^ cells per gram of sample, respectively (Fig. 3B).

Once we identified our cells of interest in the red channel, we measured the green fluorescence of those cells to determine promoter expression levels (Fig. 3A). We set an minimum threshold of 500 events, below which we would not consider a sample for analysis. This threshold ensured we only considered samples where enough data was collected in our analysis. Due to the reduced level of colonisation in the small intestine, only samples from one or two mice per promoter met the threshold for analysis for that section. However, promoter expression was quite consistent across the gastrointestinal tract (Fig. 3C and Additional file 1: Fig S2), including across the small intestine samples that could be analysed.

Promoters P_*HXT7*_ and P_*TDH3*_ were found to be the strongest across the gastrointestinal tract (Fig. 3C and Additional file 1: Fig. S3); however, both experienced high levels of inter-animal variability (Fig. 3C). Promoter P_*TEF1*_ had lower mean normalised RFI, but their expression was less variable between animals. Similarly, promoters P_*SSA1*_ and P_*HSP26*_ had similar mean expression; however, P_*SSA1*_ exhibited much lower variation between animals (Fig. 3C). P_*ALD6*_ and P_*TPI1*_ had lower overall expression but it is relatively consistent across the gastrointestinal tract, as well as having a low level of variability between animals (Fig. 3C).

### Correlation between in vitro* and *in vivo* results*

Lastly, we wanted to identify which of the in vitro characterisation set-ups best described the conditions in vivo. To do this we performed a correlation analysis comparing the normalised expression under each in vitro condition to the normalised expression in the large intestine.

**Fig. 4:**
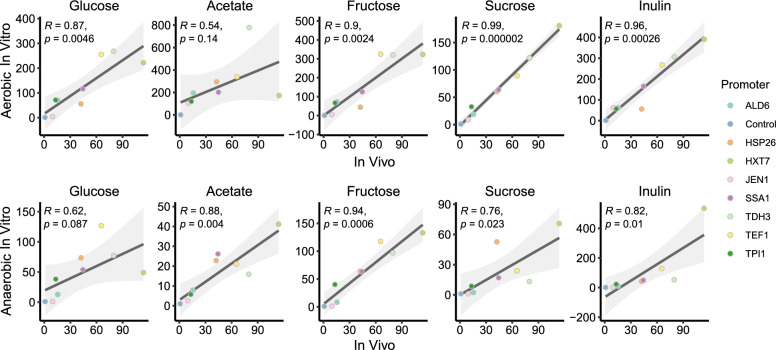
Correlation of in vitro and in vivo normalised median RFI values. Pearson correlation was used to analyse the data. P-values are adjusted for 8- and 24 h comparisons using the false discovery rate method

The correlation analysis showed that there was a high degree of correlation between the in vitro and in vivo conditions. Overall aerobic conditions showed a greater degree of correlation than anaerobic ones (average *R*-value of 0.819 and 0.798, respectively) and the 8 h time point showed a greater degree of correlation than the 24 h time point (average *R*-value of 0.828 and 0.765, respectively). As for the effect of the carbon source, fructose had the strongest correlation across time points and oxygen conditions (Fig. 4 and Additional file 1: Fig S3) and acetate had the lowest (average *R*-value of 0.908 and 0.535, respectively). However, the in vitro condition that best described the in vivo expression levels was aerobic characterisation in sucrose, at 8 h (*R*-value of 0.99).

## Discussion

*S. boulardii* has been identified as a prospective AMT chassis [6, 8, 38]; however a lack of knowledge on the translatability of in vitro promoter characterisations to in vivo applications is limiting the development of more complex *S. boulardii*-based AMTs. In this study, we characterise *S. boulardii* promoters under a variety of in vitro conditions, as well as in vivo and compare the translatability of each set of in vitro conditions.

We first characterised promoters in vitro with conventional characterisation conditions. We found that the relative expression levels of the selected promoters correlated with previously published findings [13, 14, 16]. One exception was P_*ALD6*_; Durmusoglu et al. [6] previously reported higher than expected expression from this promoter when they characterised the *S. cerevisiae* sequence in *S. boulardii*. However, our study found expression from the *S. boulardii* P_*ALD6*_ sequence to be in line with previous studies on expression in *S. cerevisiae*. Furthermore, we developed a protocol for anaerobic characterisation and found P_*DAN1*_ to be induced under anaerobic conditions, supporting that our protocol worked. While the level of induction is significantly lower than what has previously been reported (15-fold, as opposed to 300-fold) [39], we suspect it is a consequence of the differences in cultivation and characterisation method used in the respective studies.

Next, we characterised the promoters in alternative carbon sources associated with the gastrointestinal tract, diet or probiotic formulations. We found that carbon source has a significant effect on promoter expression, even for promoters generally considered constitutive. Nonetheless, we found that relative promoter expression was somewhat consistent, with strong promoters tending towards being strong in all conditions. This reflects previous findings that showed expression of *S. cerevisiae* promoters scales across conditions [19].

Our in vivo characterisation showed that colonisation is lowest in the small intestine, confirming previous findings [37]. Additionally, it showed that the same promoters express at similar levels across the gastrointestinal tract; however, promoters P_*HXT7*_, P_*TDH3*_ and P_*HSP26*_ had high levels of inter-mouse variation. Promoters P_*TEF1*_ and P_*SSA1*_ may be better choices than P_*HXT7*_, P_*TDH3*_ and P_*HSP26*_ due to their lower inter-animal variability in this study.

Comparing the promoter expression in vivo to each of the in vitro expression profiles we found that with the exception of acetate conditions, there was generally a high degree of correlation between the relative expressions of promoters in vitro and in vivo. We found that the correlation was generally stronger under aerobic conditions than anaerobic ones. This may seem contradictory given the specific inclusion of anaerobic conditions in this study due to the low-oxygen nature of the gastrointestinal tract; however the gastrointestinal tract has a radial oxygen gradient, with higher oxygen levels at the tract walls [23, 40]. Furthermore, a previous study characterising *S. cerevisiae* promoters under microaerobic conditions found a distinct microaerobic-specific expression profile [17]. Taken together, these factors could explain why anaerobic characterisation did not result in significantly greater correlation.

A limitation of this study is that our method determines the degree of correlation between relative promoter strengths. This makes our method ideal for determining which promoters to select when balancing the expression of components in complex biosynthetic pathways or biosensor circuits, but not necessarily for predicting in vivo therapeutic production levels. Furthermore, while we encourage others to characterise their promoters of interest under the in vitro conditions we have laid out, we accept that these characterisations are unable to predict the level inter-animal variability that can be determined by performing the in vivo characterisation. Finally, we selected the most common mouse strain and diet combination for our in vivo characterisation. Other mouse strains and diet combinations may not exhibit the same levels of correlation; however, due to the number of animal and diet combinations that may be of interest it is outside the scope of this study to test them all.

## Conclusion

We characterized twelve *S. boulardii* promoters and found a high degree of similarity to their *S. cerevisiae* counterparts. Our study revealed carbon-source-dependent changes in promoter expression, with P_JEN1_ showing particularly strong carbon induction. In addition, promoter expression was generally lower in anaerobic conditions, except for P_DAN1_, which exhibited higher expression under anaerobic conditions. Notably, inulin emerged as a favourable carbon source, promoting predominantly positive effects on *S. boulardii* promoter expression and may be a relevant prebiotic to investigate in future *S. boulardii* AMT tests. Overall, we hope that this study provides valuable insights into *S. boulardii* promoter characteristics, aiding in the selection of reliable promoters for targeted therapeutic applications.

## Methods

### Promoter library selection

Promoters were selected following a literature search. Selected promoters have been broadly utilised for synthetic biology, or have properties that suggest their suitability for in vivo gene expression. A full list of promoters and the rationale behind their selection can be found in Table 1.

### Media

For transformations *S. boulardii* was cultured in yeast-peptone-dextrose (YPD) medium (10 g/L yeast extract, 20 g/L casein peptone and 20 g/L glucose (Sigma Aldrich)). Transformations were streaked on synthetic complete medium without uracil plates (SC U-; 6.7 g/L yeast nitrogen base without amino acids, 1.92 g/L Yeast Synthetic Drop-out Medium without uracil, 10 g/L agar and 20 g/L glucose (Sigma Aldrich)) or SC U- supplemented with geneticin (1.7 g/L yeast nitrogen base without amino acids and ammonium sulphate, 1 g/L monosodium glutamate, 1.92 g/L Yeast Synthetic Drop-out Medium without uracil, 200 mg/L geneticin (G418), 10 g/L agar and 20 g/L glucose (Sigma Aldrich)). In vitro characterisations were cultured in synthetic complete (6.7 g/L yeast nitrogen base without amino acids, 1.6 g/L Yeast Synthetic Drop-out Medium without leucine and 76 mg/L leucine) with one of the following carbon sources as appropriate 20 g/L glucose, 20 g/L fructose, 19 g/L sucrose, 16 g/L inulin or 32.4 g/L sodium acetate (Sigma Aldrich). *E. coli* was cultured in LB supplemented with 100 mg/L ampicillin sodium salt (Sigma Aldrich). All cultures were incubated at 37 °C and 250 rpm.

### Strain and plasmid construction

Plasmids, strains, primers, and sequences used in this study are listed in Additional file 2: Tables S2, S3, S4 and S5. All oligonucleotides and double-stranded DNA fragments (gBlocks) were ordered from Integrated DNA Technologies (IDT). yEGFP was assembled into p2909 by User cloning [41], further assemblies were performed with Gibson Assembly [42] and both transformed into One Shot^®^ TOP10 *Escherichia coli* (Thermo Fisher Scientific).

*S. boulardii* with a uracil auxotrophy was used for as a base for all strains and obtained from previous work [37]. *S. boulardii* was transformed according to the protocol in Durmusoglu et al. [6]. Genomic integration cassettes were digested with restriction enzyme NotI (FastDgiest Enzyme, Thermo Scientific^™^) prior to transformation. Markerless plasmids where co-transformed with pCfB6920, into strains previously transformed with pCfB2312. Genomic integration was confirmed using colony-PCR with Taq polymerase (Ampliqon). Primers flanking the integration were used to confirm the integration. Genomic DNA was extracted by boiling cells at 95 °C for 20 min in 20 mM NaOH. One single amplification band, ~ 4000 bp, on gel electrophoresis indicated a successful integration into both chromosomes. Where necessary, strains were cured for pCfB2312 and pCfB6920 after genome integration.

### In vitro* characterisation*

Strains were streaked from − 80 °C cryostocks on to YPD plates and incubated aerobically at 37 °C for 48 h. This was repeated once, incubating anaerobically where appropriate. Experimental and pre-cultures were grown in 250 µL media in a 96-deep-well plate. Pre-cultures were inoculated with a single colony, in triplicate and incubated overnight. Experiment cultures were inoculated at an OD600 nm of 0.2. For plate reader experiments, cells were incubated in a Synergy H1 Microplate Reader (BioTek) for 48 h at 37 °C with continuous shaking and the following setting for measuring yEGFP: excitation 485/20, emission 528/20, gain 80. Growth rate analysis was performed using the Qurve app [43]. For flow cytometry experiments, at the relevant time points, 20 µL of experiment culture was diluted in 180 µL experiment solution (1X filtered PBS, 100 µg/mL cycloheximide and 2.5% DMSO) in a clear, flat-bottom microplate and incubated aerobically on the benchtop for 20 min. Flow cytometry was performed using a Novocyte Quanteon^™^ (Agilent). The following settings were used: FSC and SSC were measured with gain 400; GFP was measured using a blue laser at 525 nm and with gain 470; an FSC-H threshold of 8000 was used. Fluorescence data was collected from 2000 cells falling in the yeast gate for each sample and analysed using FlowJo software. All anaerobic work was performed using a Whitley A95 anaerobic workbench and anaerobic conditions were maintained for incubation using the BD GasPak system.

### In vivo* characterisation*

All animal experiments were conducted according to the Danish guidelines for experimental animal welfare, and the study protocols were approved by the Danish Animal Experiment Inspectorate (license number 2020-15-0201-00405). The study was carried out in accordance with the ARRIVE guidelines [44].

All in vivo experiments were performed on male C57BL/6NTac mice (6 weeks old; Taconic Bioscience). All mice were housed at room temperature on a 12 h light/dark cycle and given ad libitum access to water and a standard chow diet (Safe Diets, A30). All mice had 1 week of acclimatisation prior to antibiotic treatment and randomised according to body weight. The researchers were blinded in all mouse experiments.

Drinking water was supplemented with an antibiotic cocktail containing 0.3 g/L ampicillin sodium salt, 0.3 g/L kanamycin sulfate, 0.3 g/L metronidazole, and 0.15 g/L vancomycin hydrochloride for the duration of the study [37]. Following 5 days antibiotic treatment, mice were administered ~ 10^8^ CFU *S. boulardii* via intragastric gavage for 5 days. Mice were divided into 9 groups (n = 3), receiving either the control strain or a yEGFP expressing strain. Mice were euthanized by cervical dislocation following a 48 h wash-out period, followed by collection of gut content. Gut content was collected in pre-weighed 1.5 mL Eppendorf tubes containing 1 mL of 1 × PBS, 50% glycerol and 10 µg/mL cycloheximide and weighed again to determine content weight. All samples were kept on ice prior to treatment. The samples were homogenised by vortexing at ~ 2400 rpm for 20 min, then spun down at 100 g for 30 s. 20 µL of supernatant was added to 180 µL 1X PBS in a clear, flat-bottom microplate and transferred to the flow cytometer within 45 min of dissection. Flow cytometry gates were determined as follows: one gate was created in the red channel based on the red population present in faeces samples collected on day 1 and 4 of the study. The mKate2 + population was subsequently gated for single cells by comparing the front scatter area vs front scatter height, in order to exclude potential debris collected in the mKate2 + gate, as well as exclude double cells which could produce a higher yEGFP level than equivalent single cells. The median fluorescence of the singlets population was taken as the fluorescence read that sample (Additional file 1: Fig S4). The same gates were applied to every sample regardless of gut section.

### Statistical testing

Statistical analyses were performed in RStudio version 2023.06.0 with the tidyverse, rstatix and DescTools packages. The false discovery rate method was used to correct for multiple comparisons and the statistical significance level was set at *p* < 0.05.

### Supplementary Information

**Additional file 1: Figure S1.** Acetate, fructose sucrose and inulin 24 hrs. Normalised median relative fluorescence intensity of yEGFP from the 24-hour time point, aerobically (blue) and anaerobically (green). Bars represent the mean of three biological replicates from independent pre-cultures. Dots represent individual replicates. Error bars represent the standard deviation. Equivalent data from cultivation with glucose can be found in figure 1E. **Figure S2.** Expression in the small intestine. Dots represent individual replicates. Where there is more than one replicate present, bars represent the mean of the replicates. Error bars represent the standard deviation. **Figure S3.** Correlation at 24 hrs. Pearson correlation was used to analyse the data. P-values are adjusted for 8- and 24-hour comparisons using the false discovery rate method. **Figure S4.** Representative images of the gating strategy for the in vivo characterisation. (A) the mKate2+ cells are gated in the red channel. (B) Singlets are gated from the mKate2+ subpopulation. (C) The median yEGFP fluorescence is taken from the Singlets subpopulation in the green channel. The same gates were applied to all samples included for in vivo characterisation.

**Additional file 2: Table S1.** Growth Rates. **Table S2.** Plasmids used in this study. **Table S3.** Strains used in this study. **Table S4.** Primers used in this study. **Table S5.** Sequences of Promoters and Reporter Genes used in this study.

## Data Availability

All data generated or analysed during this study are included in this published article [and its Additional files].
