# Supplementary material for: Saccharomyces boulardii promoters for control of gene expression in vivo
Source: Microb Cell Fact. 2024 Jan 7;23:16. doi: 10.1186/s12934-023-02288-8 (PMC10771652; doi:10.1186/s12934-023-02288-8)
Supplement: Supplementary file 1 — Additional file 1: Figure S1. Acetate, fructose sucrose and inulin 24 hrs. Normalised median relative fluorescence intensity of yEGFP from the 24-hour time point, aerobically (blue) and anaerobically (green). Bars represent the mean of three biological replicates from independent pre-cultures. Dots represent individual replicates. Error bars represent the standard deviation. Equivalent data from cultivation with glucose can be found in figure 1E. Figure S2. Expression in the small intestine. Dots represent individual replicates. Where there is more than one replicate present, bars represent the mean of the replicates. Error bars represent the standard deviation. Figure S3. Correlation at 24 hrs. Pearson correlation was used to analyse the data. P-values are adjusted for 8- and 24-hour comparisons using the false discovery rate method. Figure S4. Representative images of the gating strategy for the in vivo characterisation. (A) the mKate2+ cells are gated in the red channel. (B) Singlets are gated from the mKate2+ subpopulation. (C) The median yEGFP fluorescence is taken from the Singlets subpopulation in the green channel. The same gates were applied to all samples included for in vivo characterisation. [file 12934_2023_2288_MOESM1_ESM.docx]

**Supplementary Figures**

**
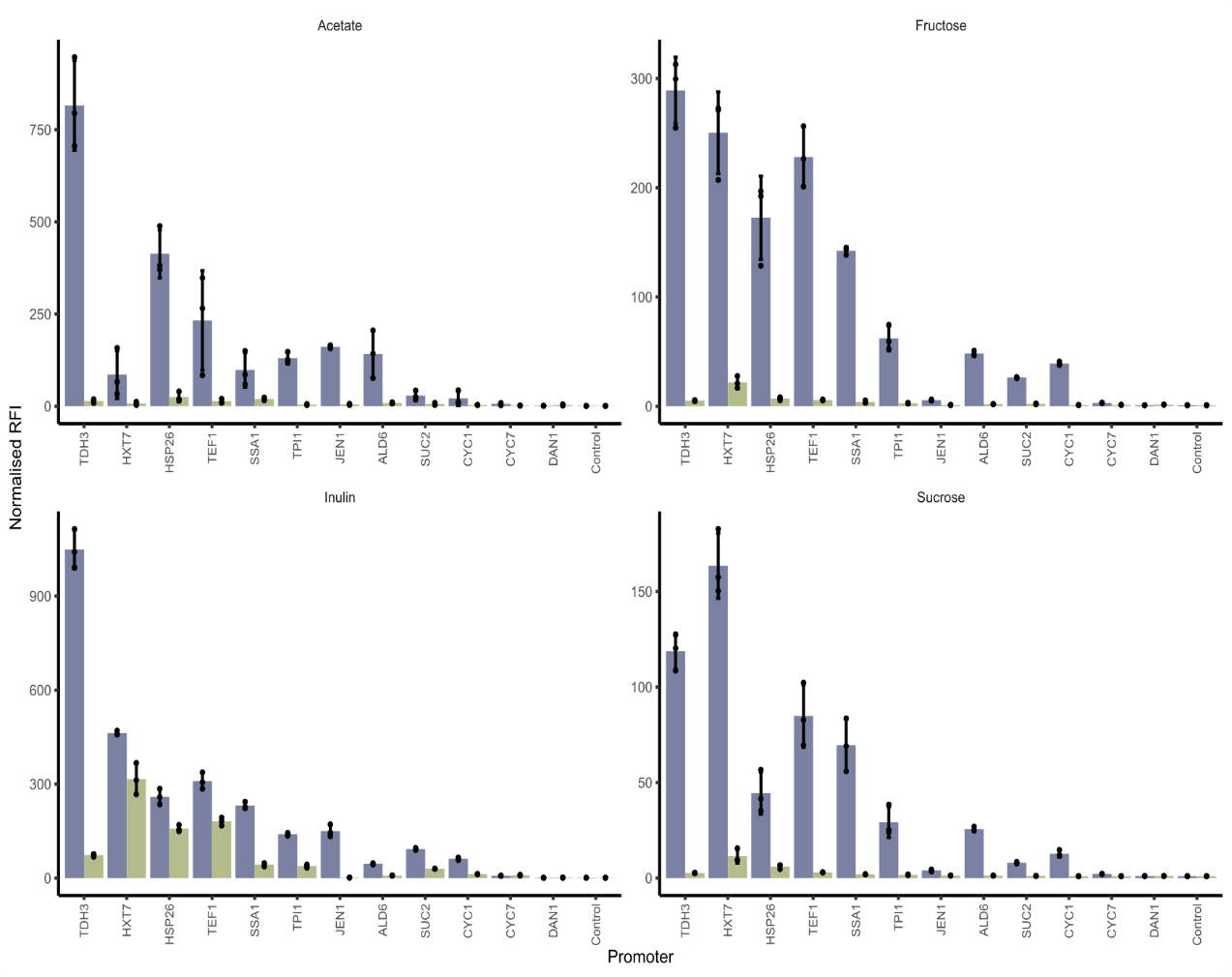
**

**Figure S1: Acetate, fructose sucrose and inulin 24 hrs.** Normalised median relative fluorescence intensity of yEGFP from the 24-hour time point, aerobically (blue) and anaerobically (green). Bars represent the mean of three biological replicates from independent pre-cultures. Dots represent individual replicates. Error bars represent the standard deviation. Equivalent data from cultivation with glucose can be found in figure 1E.

**
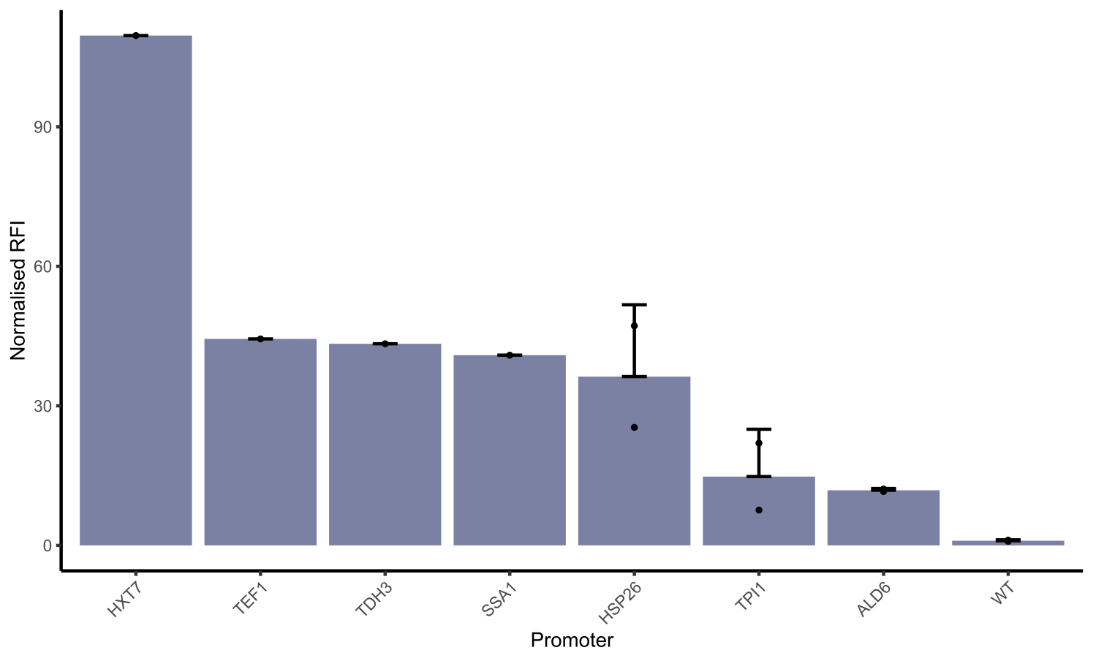
**

**Figure S2: Expression in the small intestine.** Dots represent individual replicates. Where there is more than one replicate present, bars represent the mean of the replicates. Error bars represent the standard deviation.

**
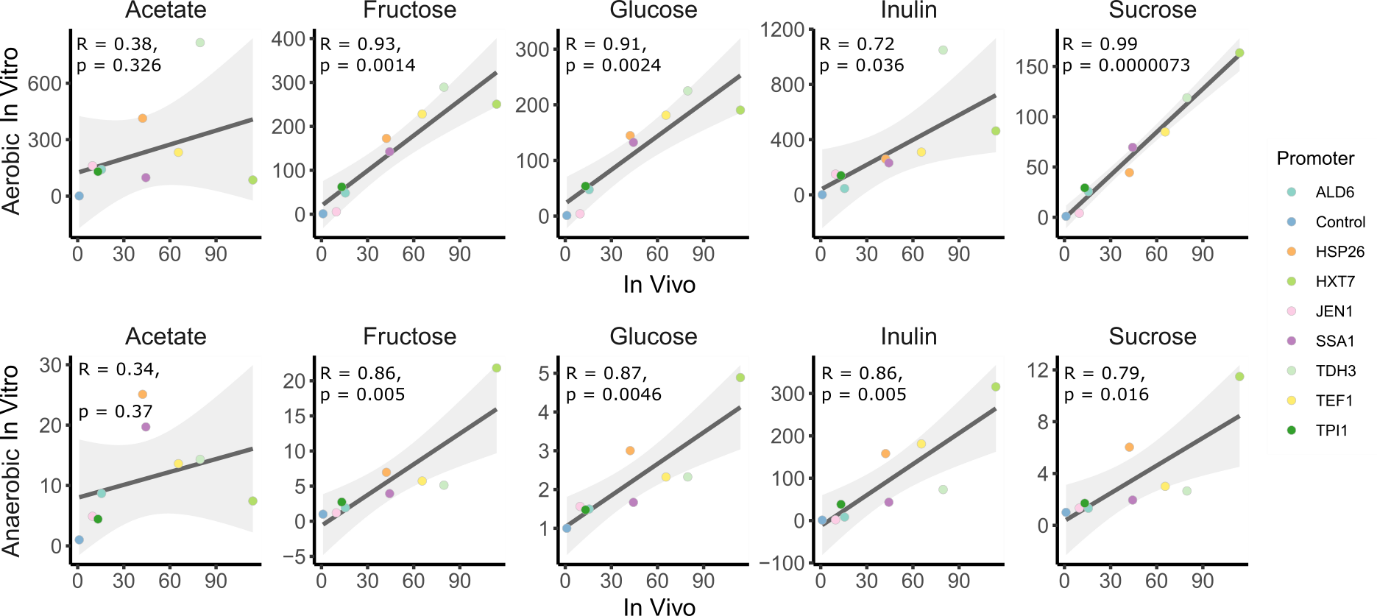
**

**Figure S3:** **Correlation at 24 hrs.** Pearson correlation was used to analyse the data. P-values are adjusted for 8- and 24-hour comparisons using the false discovery rate method.


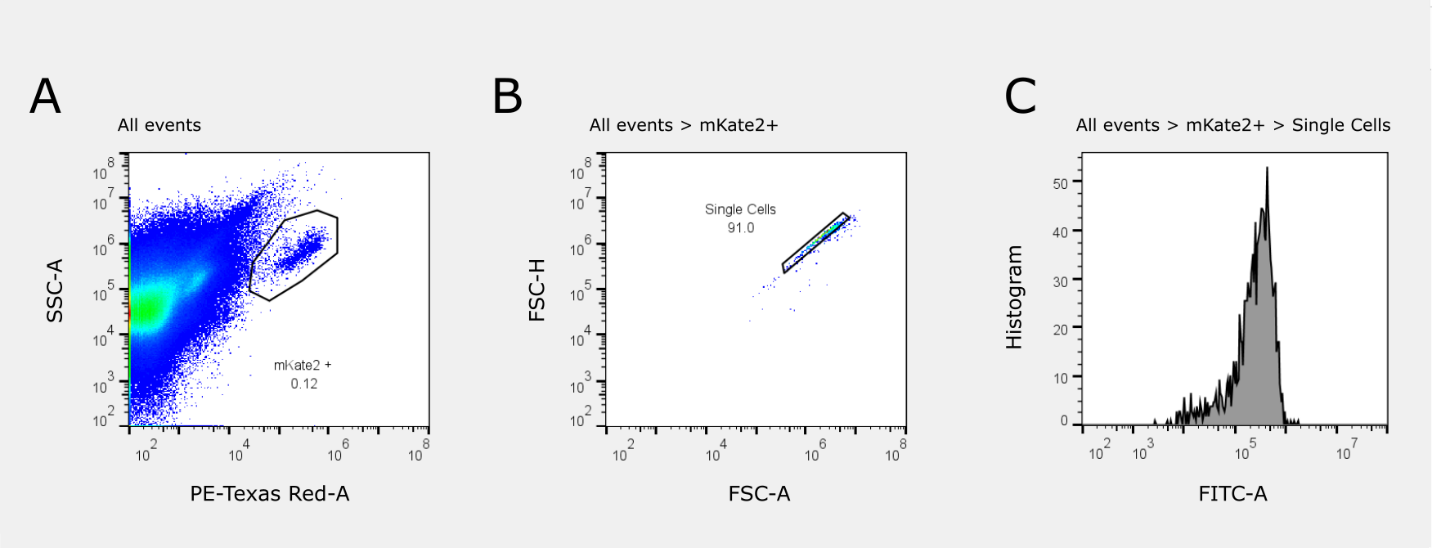


Figure S4: Representative images of the gating strategy for the in vivo characterisation. **(A)** the mKate2+ cells are gated in the red channel. **(B)** Singlets are gated from the mKate2+ subpopulation. **(C)** The median yEGFP fluorescence is taken from the Singlets subpopulation in the green channel. The same gates were applied to all samples included for in vivo characterisation.
